# Large Dog Relinquishment to Two Municipal Facilities in New York City and Washington, D.C.: Identifying Targets for Intervention

**DOI:** 10.3390/ani4030409

**Published:** 2014-07-08

**Authors:** Emily Weiss, Margaret Slater, Laurie Garrison, Natasha Drain, Emily Dolan, Janet M. Scarlett, Stephen L. Zawistowski

**Affiliations:** 1 Shelter Research and Development, Community Outreach, American Society for the Prevention of Cruelty to Animals (ASPCA^®^), 3201 SW Winding Way, Palm City, FL 34990, USA; 2 Shelter Research and Development, Community Outreach, American Society for the Prevention of Cruelty to Animals (ASPCA^®^), 50 Stone Ridge Drive, Florence, MA 01062, USA; E-Mail: margaret.slater@aspca.org; 3 Shelter Research and Development, Community Outreach, American Society for the Prevention of Cruelty to Animals (ASPCA^®^), P.O. Box 408, Little Silver, NJ 07739, USA; E-Mail: laurie.garrison@aspca.org; 4 Shelter Research and Development, Community Outreach, American Society for the Prevention of Cruelty to Animals (ASPCA^®^), P.O. Box 4323, Arlington, VA 22204, USA; E-Mail: natasha.drain@aspca.org; 5 Shelter Research and Development, Community Outreach, American Society for the Prevention of Cruelty to Animals (ASPCA^®^), P.O. Box 821075, Kenmore, WA 98028, USA; E-Mail: emily.dolan@aspca.org; 6 Department of Population Medicine and Diagnostic Sciences, College of Veterinary Medicine, Cornell University, Ithaca, NY 14853, USA; E-Mail: jms15@cornell.edu; 7 American Society for the Prevention of Cruelty to Animals (ASPCA^®^), 520 Eighth Avenue, 7th Floor, New York, NY 10018, USA; E-Mail: stephen.zawistowski@aspca.org

**Keywords:** dog relinquishment, animal shelter, community interventions, safety net

## Abstract

**Simple Summary:**

While the overall trend in euthanasia has been decreasing nationally, large dogs are at a higher risk of euthanasia than other-sized dogs in most animal shelters in the United States. We hypothesized that one way to increase the lives saved with regard to large dogs in shelters is to keep them home in the first place when possible. Our research is the first to collect data in New York City and Washington, D.C., identifying the process leading to the owner relinquishment of large dogs. We found that targets for interventions to decrease large dog relinquishment are likely different in each community.

**Abstract:**

While the overall trend in euthanasia has been decreasing nationally, large dogs are at a higher risk of euthanasia than other sized dogs in most animal shelters in the United States. We hypothesized one way to increase the lives saved with respect to these large dogs is to keep them home when possible. In order to develop solutions to decrease relinquishment, a survey was developed to learn more about the reasons owners relinquish large dogs. The survey was administered to owners relinquishing their dogs at two large municipal facilities, one in New York City and one in Washington, D.C. There were 157 responses between the two facilities. We found both significant similarities and differences between respondents and their dogs from the two cities. We identified opportunities to potentially support future relinquishers and found that targets for interventions are likely different in each community.

## 1. Introduction

Millions of dogs enter the shelter system each year, and in many cases, over half of those dogs are relinquished by their owner [1]. Large dogs are often at a higher risk of euthanasia than other dogs in the sheltering system. Work done by Brown *et al.* [2] indicated that the average length of stay in shelters for medium-sized and large dogs was significantly longer than for extra-small and small dogs. Longer lengths of stay can result in both strain on shelter resources and negative health and behavior outcomes for the dogs. Large dogs are also less likely to be adopted [3] and to leave shelters alive [4]. Size is also an issue in that there are and may be additional legislative or housing restrictions based on the sizes of pets [5] and certain large breeds or breed mixes, precluding people from owning a large dog. This can both precipitate relinquishments and discourage potential adopters from adopting large dogs. 

Numerous studies have been published regarding the characteristics of dogs relinquished to animal shelters and the people that relinquish them. Most of these studies were conducted in the 1990s and early 2000s, and none evaluated whether the size of dog influenced the reasons for relinquishment. In those studies, the characteristics of the dogs associated with a higher risk of relinquishment included being of mixed breeding [6], older than five months [6,7] and reproductively intact [6,7,8]. These dogs were more likely to be acquired at low or no cost and to have been acquired from friends, shelters, pet shops or had been a stray [7]. Relinquished dogs were also less likely to receive obedience training or veterinary care [6,9]. 

The characteristics of people and their families that put them at higher risk of relinquishing a dog included age (e.g., younger adults) [5,6,7], lower annual income (less than $20,000–$30,000) [5,8] and lower overall educational achievement [6,7]. Many who relinquished dogs lived in rental properties [8] or were not living alone in the home [5,8]. Owners relinquishing their dogs were also more likely to have misconceptions about their animals, housetraining, the cost of maintenance and the timing of neutering [7]. Unrealistic expectations are noted as one of the strongest risks to breaking the bond between humans and companion animals [10]. Those relinquishing pets for behavior reasons seem, at least in one study [11], to make misattributions or to over-generalize from the ‘failed adoption’. When relinquishing a dog for behavior issues, adopters reported that they would choose their next dog to be a younger, or older, or smaller, or different breed of dog. This is powerful, as it reveals the unrealistic expectations of the relinquisher (for example, he is a small dog and, therefore, behaviorally sound).

Certainly, dogs of any breed can be relinquished, and most are mixed breeds [6]. In some places, such as California, certain breeds, such as German shepherds, chows, Labrador retrievers, Staffordshire terriers, rottweilers and cocker spaniels, are more likely to be relinquished [12]. However, some breeds may be more likely to be relinquished. For example, one study found that pit bull-type dogs were more common in one relinquished group when compared to those visiting a vaccination clinic, yet neither group was more likely to report behavior problems [13]. In the above study, breeds may be misrepresented based on how the vaccination clinic was marketed and the availability and accessibility of the clinic. 

Less research is available evaluating the process leading to relinquishment (e.g., timing and options considered). DiGiacomo *et al.* [14] found that the decision to relinquish animals was not sudden for many owners; in fact, the process lasted weeks to months for many people. The reasons for relinquishment in that study generally fell within the themes of moving and/or landlord issues and behavioral problems. They found that people tended to tolerate problems associated with the dog until those problems exceeded some unmeasured decision-making threshold. In the same study, researchers reported that some people attempted to resolve the issues themselves (such as attempts to rehome the dogs), but were poorly informed about how to find solutions. Shore *et al.* 2003 [5] also reported that owners attempted to rehome their dogs via personal contacts and found that some relinquishers had high levels of emotional involvement with the animals that were being relinquished. They also reported that many low-income renters had to move for employment reasons. They found that landlord restrictions were an important factor, either because dogs were forbidden in rental housing, the renter already had too many animals or the dogs were too large.

Little is also known about the activities and types of programs that could assist owners before they relinquish their dogs. One exception is a pilot intervention instituted by The Downtown Dog Rescue in South Los Angeles, targeting an economically underserved population with high dog intake and euthanasia rates. They offered owners resources and support, including veterinary care, training, food and yard fencing, to help people keep their dogs. During the intervention period, 91% of potentially relinquished dogs were kept out of the shelter, because the owner was able to take advantage of the intervention services and keep their dog. Almost two thirds of those services involved free spaying/neutering and services to assist with housing problems [15]. 

The present study was designed to study the factors leading to and surrounding the relinquishment of large dogs to shelters in two large eastern cities, including dog and owner demographic data. Specifically, the objectives were to identify: (1) the original reasons for the acquisition of the dog and any reservations owners may have had at that time; (2) what may have changed to precipitate the relinquishment and other reasons for relinquishment; (3) options that owners had explored before relinquishment and the timing of their deliberations; (4) owners’ perceptions of resources or support that might have helped them retain their pet; and (5) whether the dog and owner demographics or the responses mentioned in Objectives 1–4 varied across two communities. The ultimate intention was to develop a sufficient understanding of the relinquishment of large dogs for which interventions can be developed in these two (and possibly other) communities to decrease the numbers of large dogs relinquished to shelters.

## 2. Experimental Section

### 2.1. Participating Shelters

The study was conducted using interviews with owners relinquishing large dogs at two large municipal facilities in the Eastern United States, the Washington Humane Society in Washington, D.C. (DC), and the New York Animal Care and Control Bronx Pet Receiving Center in the Bronx, NY (NYC). These two facilities were chosen as a convenience sample, since they were both large urban facilities in which the American Society for the Prevention of Cruelty to Animals (ASPCA) had relationships needed to conduct the research. The NY ACC had an overall live release rate (live releases/intake) for canines at the time of the study of 77.6% [16]; and in DC, the live release rate was 68.1% at both Washington Humane Society facilities [17]. 

### 2.2. Survey Participants

The surveys were conducted in person by two similarly-trained temporary contractors to the ASPCA during April to August, 2012, in DC and August to March, 2013, in NYC. The interviewers were in the shelters during open hours. The hours varied throughout the study period to best capture busy relinquishment times as they were discovered. They approached those relinquishing dogs weighing an estimated 40 pounds or more, with contractors instructed to use their best judgment as to the weight. Owners with dogs that appeared to be under 4 months of age and puppies relinquished without a bitch were not approached. Relinquishers were approached after they completed the relinquishment paperwork and the dog was handed over to the intake staff. Two screening questions were initially asked of potential participants who had a dog that appeared to be at least four months of age and at least 40 lbs: whether the dog belonged to the respondent or a family member; and whether they owned the dog for less than two weeks. Only people relinquishing a dog(s) belonging to that person (or their family) and who had owned the dog for two weeks or more were included. Participants were told that the survey was being conducted by the ASPCA. These criteria were used to ensure that the person completing the survey had adequate knowledge about the family situation and the dog to provide accurate answers.

Since the lobbies of these shelters were busy and the interviewers were not always present when the shelter was open, it was not possible to accurately assess the percentage of large dogs that were successfully enrolled.

### 2.3. Survey Design

The survey consisted of 26 questions, five of which were demographic questions regarding income, education, number of children and adults in the household, type of dwelling and ethnic identity. Income data were collected for the household; educational achievement was collected for the person with the highest level of education in the household; ethnic identity was collected as reported by the respondent. When reporting ethnic identity, the majority of respondents chose one of the available categories. The few people who reported more than one identity were included in the “other” category. Five questions inquired about characteristics (including age, gender, spay/neuter status, purebred status and, if purebred, what breed) of each dog. Since ‘bully-type’ (the category of bully-type dogs commonly include pit bull-type, American bull dog-type and similar build dogs) dogs are often more at risk in shelters, breed was also categorized into two groups: bully-type and non-bully-type. The determination was made visually by the contractor administering the survey and included dogs identified by their owners as pit bulls. Other questions addressed how often the dog had been to a veterinarian, his/her cost of acquisition, source (e.g., friend, shelter), length of ownership, reason(s) for original acquisition, attributes the dog possessed that led to his/her selection, characteristics that the owners liked best about the dog in the first few months in the household and about any reservations they may have had about getting the dog initially. The remaining questions pertained to changes in the household that contributed to the relinquishment, reasons for the relinquishment, actions taken to keep the dog in the home, those involved in the decision to relinquish the dog, the time frame during which the decision to relinquish had been considered, anything that may have helped them keep the dog and what they would like a new adopter to know. The complete survey, including instructions to the interviewer, can be found in Appendix.

### 2.4. Management of Responses

Answers to multiple-choice questions with an open-ended “other” category (to which respondents could add a response not listed among the other choices) were either incorporated into one of the given choices (where appropriate) or made into a new category. For example, responses of “family” to the question asking about where respondents had obtained their dog were combined with the “friend” responses into a friend/family category. Similarly, for yes/no questions with an option “if yes, please describe”, similar answers were grouped and given a category. For example, people answering yes to whether they had reservations when first acquiring their dog were asked to describe their reservations. Their reservations were grouped into three categories for summary and analysis: (1) *housing-related* (e.g., my building did not allow big dogs, worried about housing rules); (2) dog-related (e.g., concerned about the dog’s temperament); and (3) people-related (e.g., concerned that another person would not want the dog). Similarly, for people answering yes to whether something had changed in the household that contributed to the relinquishment, responses were summarized in five categories: people issues, moving, landlord issues, behavior issues and dog health/expenses. The people issues category included overall financial, people health, child-related and lack of time issues. Having to move or already moved were combined into the moving category, and landlord issues included responses, such as “the landlord found out about the dog”, “the landlord decided to start enforcing the no dog rule” or “the housing rules changed”. The behavior issues category included responses, such as those relating to housetraining, aggression or chewing. The dog health/expenses category included the inability to pay for veterinary care, pet food or to just care for the dog. 

Categories were also created for responses to open-ended questions. For example, responses to two questions, one asking about characteristics that led them to select their dog and the other asking what they originally liked best about the dog, were categorized into the same 4 categories as follows: appearance included responses about how the dog looked (e.g., color, cuteness, big eyes, size). The behavior with people category included responses that described how the dog interacted with people (e.g., came to me, friendly, loving), while the personality/temperament included answers, such as “well-behaved, trained, energetic or mellow”. The final other category included all other answers, such as “only one left,” “dog reminded owner of himself” or “help out a friend”.

### 2.5. Statistical Analysis

Categorical responses and questions that allowed more than one response were summarized using frequencies and percentages. Comparisons of categorical responses between the two communities, as well as comparisons of owner demographic variables (household income, education and ethnic identity) and neuter status of their pet were made using the chi-square test. In analyses of categorical data where at least one cell had an expected frequency of ≤5, the Fisher exact test was used. The number of people in households was summarized using means and medians. An alpha level of ≤0.05 was used for all statistical tests. Statistical analyses were conducted using Stata/SE 12 (StataCorp LP, College Station, TX, USA).

## 3. Results

There were a total of 157 respondents, 74 in NYC and 83 in DC. In NYC, two households brought in two dogs, resulting in a total of 76 dogs relinquished. In DC, five people brought in two dogs, resulting in a total of 88 dogs relinquished. Demographic data reflects the number of households, while dog data reflects individual dogs.

### 3.1. Household Characteristics

Household characteristics of respondents from the two communities were similar for some factors and differed considerably for others (Table 1). Although not statistically different, household income in DC was somewhat higher than in NYC. Similarly, the highest educational achievement in Washington households was slightly higher than in New York, but the overall education level was not significantly different between the two communities. The ethnic backgrounds of respondents in the two cities were statistically different. People from DC were significantly more likely to be African-American, while those in NYC were more likely to be Hispanic/Latino. The type of dwelling was significantly different between the two communities, with the majority of NYC respondents living in apartments, while a subset in DC lived in single family homes. The majority of households in both communities had at least one young child who was 12 years of age or less, and both communities had a median of three people in the households of respondents.

**Table 1:** Characteristics of respondents’ households in New York City (NYC) and Washington, D.C. (DC).

Human Demographics	NYC		DC	
Household Income ($)	No. *	%	No. *	%
<35,000	33	48.5	37	50.7
35,000–74,999	15	22.1	21	28.8
≥75,000	5	7.4	8	11.0
Don’t know	15	22.1	7	9.6
Ethnic Background **	No. *	%	No. *	%
Hispanic/Latino	41	57.7	1	1.3
African-American/black	17	23.9	68	88.3
Other	13	18.3	8	10.4
Education level	No. *	%	No. *	%
Did not complete high School	9	12.7	0	-
Completed H.S.	12	16.9	22	28.6
Some college, associate degree	23	32.4	23	29.9
bachelor’s degree	24	33.8	24	31.2
Graduate/professional degree	2	2.8	8	10.4
Do not know	1	1.4	0	-
Type of Dwelling **	No. *	%	No. *	%
Apartment or condo	58	80.6	39	50.0
Single family housing	6	8.3	23	29.5
Other	8	11.1	16	20.5
Ages of House Hold members (y)	No. *	%	No. *	%
<5	37	51.4	37	48.1
5–12	35	48.6	53	68.8
13–19	35	48.6	36	46.8
20–64	151	209.7	155	201.3
≥65	6	8.3	11	14.2

* Numbers for each characteristic may not sum to 74 (NYC) or 83 (DC) because of missing information for some households or more than one answer being possible (ages). ** *p* < 0.001.

### 3.2. Dog Characteristics

As with the household characteristics of the respondents, there were similarities and differences in the dog characteristics between the two communities (Table 2). 

#### 3.2.1. Age, Gender and Number of Dogs in the Household

As shown in Table 2, the age and gender distributions of relinquished dogs were not significantly different. The majority of respondents had only the surrendered dog in the household and about a quarter had one other dog (26% NYC, 25% DC). One household in NYC reported that they had three other dogs.

**Table 2:** Characteristics of large dogs surrendered to shelters in New York City and Washington, D.C.

Dog characteristics	NYC		DC	
Age Group (y)	No. *	%	No. *	%
<1	18	23.7	25	28.4
1–5	37	48.7	44	50
>6	21	27.6	17	19.3
Breed Group **	No. *	%	No. *	%
Bully-type	61	80.2	50	56.8
Non-bully-type	15	19.8	38	43.2
Veterinary Visits **	No. *	%	No. *	%
0	31	40.8	33	37.5
1	20	26.3	52	59.1
≥2	21	27.6	2	2.3
Don’t know	4	5.3	1	1.1
Purebred **	No. *	%	No. *	%
Yes	34	44.7	5	5.7
No	35	46.1	83	94.3
Do not know	7	9.2	0	-
Neutered **	No. *	%	No. *	%
Yes	25	32.9	16	18.2
No	50	65.8	72	81.8
Do not know	1	1.3	0	-
Gender	No. *	%	No. *	%
Male	46	60.5	52	59.1
Female	30	39.5	36	40.9

* Numbers for each characteristic may not sum to 76 (NYC) or 88 (DC) because of missing values. ** *p* < 0.05.

#### 3.2.2. Neutering, Age at Neutering and Visits to the Veterinarian

There were some key differences in spay/neutering across the communities. While the rate of spaying/neutering was not different by gender, a significantly smaller proportion of dogs in DC had been spayed or neutered compared to NYC. Neuter rates did not differ by household income, education or ethnic identity in either community. 

In NYC, 15% of respondents reported “do not know” when asked when the dog was neutered, with the rest reporting six months of age or younger (36%), between the ages of seven and 12 months (32%) and 16% after one year. Most people in DC responded “do not know” when asked when the dog was neutered (88%), and the remainder reported that the dog was neutered at six months or under (no statistical tests were run, due to the small sample size of each group).

Respondents from the two communities differed significantly in the use of veterinary care for their dogs (Table 2). While slightly more DC dogs had at least one veterinary visit, dogs of respondents in NYC had two or more veterinary visits significantly more often than dogs of respondents in DC.

#### 3.2.3. Reported Purebred Status and Breed

Respondents in NYC were significantly more likely to report that their dog was purebred. Of the 34 dogs reported as purebred in NYC, 80% were reported as some form of pit bull (23% pit bull, 40% red nose pit bull, 17% blue nose pit bull). Significantly more bully-type breeds (as determined by visual identification by the interviewer) were relinquished by respondents in NYC than DC. 

### 3.3. Ownership Characteristics

As shown in Table 3, when asked how much the dog cost to obtain, most respondents paid nothing, while 16% in both communities reported paying over $300 for their dog. Most people obtained their dog from a friend, family member or acquaintance. The remaining dogs were obtained from a wide variety of sources, including breeders, strangers or shelters. The other category included a pet store, born in the home, received as a gift and found as a stray. It is interesting to note that few people obtained their dog at an animal shelter. The length of ownership was similar in both cities, with the majority of owners having the dogs for more than one year. None of these characteristics were significantly different between the two locations.

**Table 3:** Ownership characteristics of large dogs relinquished to a shelter in New York City and Washington, D.C.

Ownership Characteristics	NYC		DC	
Cost of dog	No *	%	No *	%
$0	51	67.1	54	61.4
$0.01 to <$300	12	15.8	19	21.6
>$300	12	15.8	14	15.9
Where did they obtain dog	No *	%	No *	%
Friend/family/acquaintance	40	52.6	49	55.7
Breeder	10	13.2	10	11.4
Stranger	8	10.5	3	3.4
Animal shelter	3	3.9	6	6.8
Other	15	19.7	20	22.7
Why did they obtain the dog **	No *	%	No *	%
Companion to child Companion to adult	23 28	30.3 36.8	37 55	42 62.5
For protection	15	19.7	10	11.4
“Just wanted a dog”	25	32.9	3	3.4
To help a friend	18	23.7	3	3.4
To save a life	14	18.4	0	-
Spur of the moment	14	18.4	0	-
Gift	8	10.5	7	8
Teach child responsibility	8	10.5	0	-
Other	23	30.3	6	6.8
Length of ownership (months)				
≤12	32	42.1	35	39.8
>13–36	18	23.7	29	33
≥37	26	34.2	24	27.3

* Numbers for each category may not sum to the total number of HH because of missing information for some households. ** Percentages may be greater than 100%, because multiple responses were allowed. This also precluded statistical analysis, since the assumption of independence would have been violated.

Respondents could choose from a list of reasons, as well as provide an open-ended answer in the interview to answer why they initially acquired their dog. As shown in Table 3, the majority of people in both communities indicated that they got the dog as a companion to a child, as a companion to an adult and/or for protection. Few reported that the dog was a gift. Respondents in NYC chose a wider variety of options than those in DC and also indicated that they wanted to help a friend, to save a life or admitted to making a spur of the moment decision (e.g., adopted because the animal was cute). NYC respondents also reported “just wanted a dog” in the open field response. The other category included responses, such as “offspring of family dog” or “first dog.” 

#### Why Did They Choose and What Did They Like Best about the Dog?

**Figure 1:**
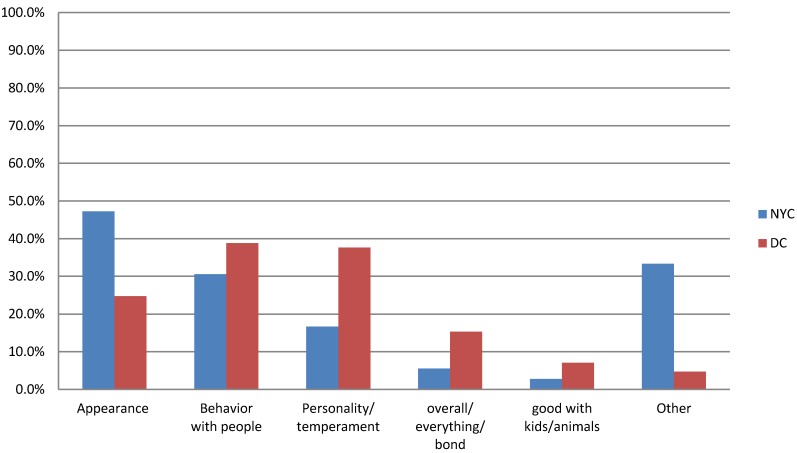
Comparison of dog characteristics that influence owner selection in New York City and Washington, D.C.

People were asked to think back and identify the characteristics that led them to select their dog (as opposed to another dog) and what they liked best about the dog back during the first few months of ownership. Multiple responses were allowed for both questions. As shown in Figure 1, the top two characteristics that initially drew respondents to their dog in NYC were appearance (47%) and behavior with people (31%), while in DC, the top two were behavior with people (39%) and personality/temperament (38%). Respondents provided a more diverse set of answers in NYC than in DC, resulting in more responses in the other category, which included “felt sorry for” or “other person chose dog”.

**Figure 2:**
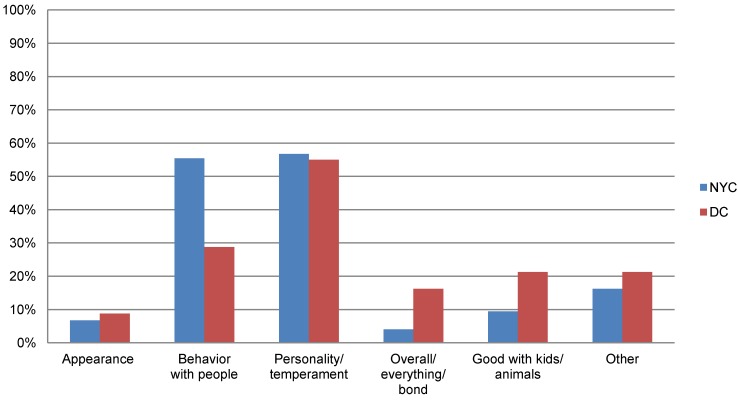
Comparison of dog characteristics most favored by owners after several months of ownership in New York City and Washington, D.C.

In contrast, once the dog was in the home, appearance became almost immaterial (Figure 2) in both communities. Rather, over half of the respondents in both communities cited personality/temperament as the characteristic they liked best during the first few months the dogs were in their homes. Interestingly, respondents in NYC cited the dog’s behavior with people as what they liked best more often compared to respondents in DC.

### 3.4. Relinquishment of the Dog

#### 3.4.1. Reservations with Regards to Acquiring the Dog Initially

Most people in both communities did not have reservations or concerns about initially acquiring their dog, but significantly more people in NYC (29%) reported that they had some reservations compared to DC (12%) (*p* = 0.009). When asked what the reservations were, there were a wide variety of answers, but 32% of responses in NYC had to do with housing concerns (“landlord does not allow pets” or “worried about housing rules”), while no housing concerns were reported in DC. In NYC, 36% of responses involved concerns about some characteristic of the dog (health, temperament, age, size) as opposed to only 9% of responses from DC. In DC, only eleven respondents identified the nature of their reservation, and they were primarily people-related: for example, how another person would react to the dog; they were not looking for another dog; or the respondent “was a cat person”.

#### 3.4.2. Changes in the Household Leading to Relinquishment

**Figure 3:**
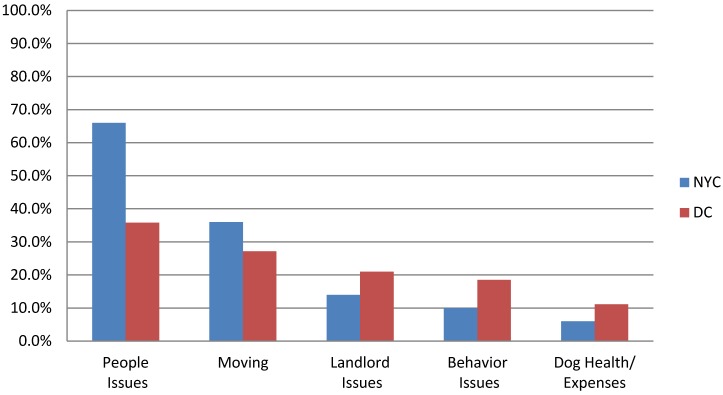
Comparison of factors that influence the relinquishment of large dogs in New York City and Washington, D.C.

When asked if anything had changed recently in the household that contributed to the decision to relinquish the dog, 68% of people in NYC answered “yes” compared with 98% of people in DC (*p* < 0.001). If a change had occurred, respondents were asked for specific reasons, and multiple responses per respondent were collected. As shown in Figure 3, a higher proportion of respondents in NYC (66%) reported changes in people-related issues (e.g., financial, child-related) than DC (36%). Interestingly, behavior issues and dog health/expenses were cited least often.

A follow-up question asked for additional reasons for relinquishing the dog other than what was reported in the above question, and up to five reasons were collected. In NYC, the majority of reasons given (57%) had to do with characteristics of the dog (behavior, aggression, expense, size). A quarter of the reasons had to do with people issues (personal finances, health, time) and 14% with housing issues (landlord issues or not enough space). In DC, only 12 responses were given. Five reasons had to do with characteristics of the dog, two with people issues and one with housing. Other responses included reasons, such as “not my dog” or “was able to find a home for the other dog.”

#### 3.4.3. Decision Factors Considered before Relinquishment

As shown in Table 4, over 60% of respondents in both communities reported deliberating for more than a week, with half of all respondents considering their decision a month or more before relinquishment. There was no significant difference between the two communities in their length of deliberations.

When asked who was involved in the decision to relinquish the dog, responses were similar in the two communities. The respondents alone, in consultation with their spouse, the entire family, family and friends or their parents alone were reported.

Significantly more people in NYC (85%) reported having explored options (other than the shelter where the dog was relinquished) before relinquishing the dog compared to those in DC (69%) (*p* = 0.03). The top five options tried in NYC were: family or friends; contacting a help line, shelter (other than the one used) or rescue; social media or ads/flyers; and giving away or trying to sell the dog. Ninety percent of people in DC said that they tried family or friends (Table 4).

**Table 4:** Decision factors considered before the dog was relinquished to a shelter in New York City and Washington, D.C.

Decision Factors	NYC		DC	
Length of deliberation (wk) ≤1 >1 to <4 ≥4	No. *	%	No. *	%
	28	37.8	27	33.3
	8	10.8	15	18.5
	38	51.4	39	48.1
Who made the decision Respondent only With partner/spouse Entire family With family/friends Parents	No. *	%	No. *	%
	35	47.3	38	45.8
	11	14.9	21	25.3
	13	17.6	9	10.8
	9	12.2	10	12
	6	8.1	4	4.8
Options considered ** Family/friends Contact helpline/shelter/rescue Giveaway/tried to sell Social media/ads/flyers Other	No. *	%	No. *	%
	42	66.7	51	89.5
	16	25.4	0	-
	9	14.3	2	3.5
	10	15.9	0	-
	16	25.4	6	10.5

* Numbers for each category may not sum to the total number of households because of missing information for some households or more than one answer was possible. ** Percentages may be greater than 100% because multiple responses were allowed. This also precluded statistical analysis, since the assumption of independence would have been violated.

#### 3.4.4. Assistance that Might Have Prevented Relinquishment

**Figure 4:**
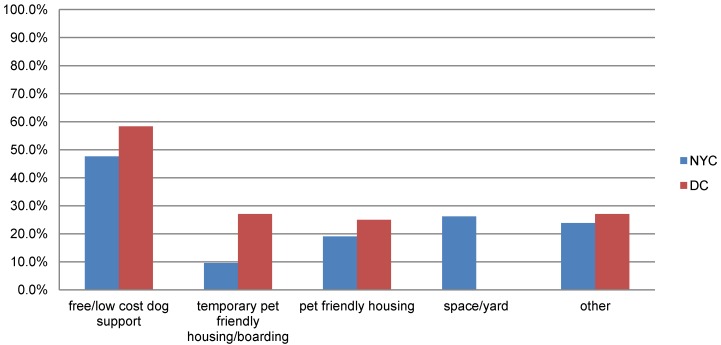
Comparison of resources, identified by relinquishers, which may have prevented surrendering of large dogs to shelters in New York City and Washington, D.C.

Interestingly, more than half of respondents in both communities (57% NYC, 58% DC) answered that some assistance may have helped them retain their dog. When asked to identify what might have helped in an open-field, multi-response format, 48% of respondents in NYC and 58% in DC said some form of low-cost or free dog support, such as training, veterinary care, day care, boarding (included in this category only if cost was specifically mentioned) or pet food. Pet-friendly housing was mentioned in both communities (19% NYC, 25% DC). In DC, 27% of people specified that temporary pet-friendly housing and in NYC 10% of respondents indicated that boarding would have helped. In NYC, 26% of people said more space (some respondents specified a larger yard or apartment; some simply responded with the need for more space) could have helped (Figure 4).

#### 3.4.5. Information about Their Dog that Respondents Would Share with a New Adopter

**Figure 5:**
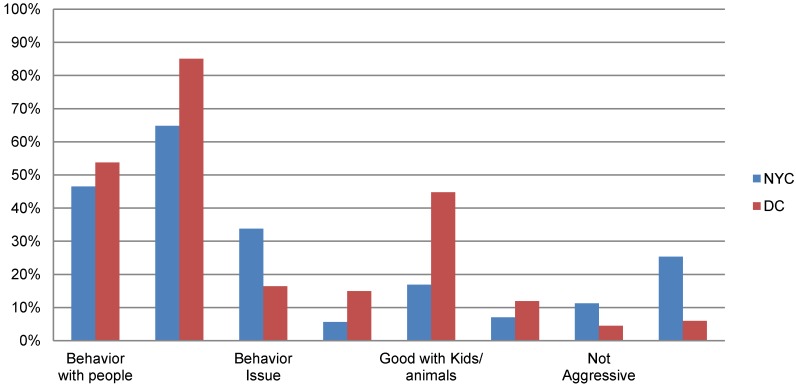
Comparison of information provided by relinquishers when asked, ‘what might help potential new adopters?’ in New York City and Washington, D.C.

This was an open-ended question, and the majority of responses had to do with the personality/temperament of the dog. The responses included “likes to play tug-of-war and be rubbed”, “does not like baths” or “loves to swim and play”. Comments relating to the dog’s behavior were the second most frequent type of comments that respondents would pass on to potential adopters. Affectionate behavior towards people was cited often, such as “likes to cuddle with me” or “very loving.” Specific behavior issues that might help new adopters avoid problems with the dog were mentioned, such as “chews on wires and wood”, “does not do well with other animals in the home” or “not housetrained” (Figure 5).

## 4. Discussion

This research examined factors associated with the relinquishment of large dogs to two large animal welfare agencies in two urban communities. We conducted this research in order to identify potential solutions for those relinquishing large dogs to shelters. Our study examined the characteristics of dogs and the people relinquishing them. Specifically, we asked people the reason for relinquishment, including how long they had been thinking about relinquishing their dog and what changed in their lives that contributed to that decision. We also explored the nature of options explored before the dog was relinquished and what, if anything, respondents believed could have helped them keep the dog in their household. By conducting the survey in two different urban communities, we hoped to assess the similarities and differences in the needs around relinquishment issues that may be community based.

The characteristics of the large dogs being relinquished at our two sites are similar to the characteristics found in other studies [6,7,8]. The majority of the dogs were over one year of age and intact. In DC, nearly all of the dogs were reported to be mixed-breed. Although 45% of dogs in NYC were reported to be purebred, the majority of those were reported to be pit-bull-type dogs. This is of interest to note, as bully-type dogs are both increasing in popularity in the pet owning population [8,18,19] and tend to enter many municipal facilities in higher numbers than other breed types. Although previous literature found that male and female dogs were relinquished in about the same proportions, the majority (60%) of the dogs in this study were male.

Similar to findings from previous studies, dogs relinquished to these two shelters were spayed/neutered at much lower rates than in the overall owned dog population. Data from the American Pet Products Association [20] show that 83% of owned dogs are spayed or neutered, that female dogs are much more likely to be neutered and that people with higher incomes are more likely to have their dog spayed or neutered. Previous studies from over a decade ago reported that 54–65% of the dogs relinquished to shelters were unaltered [6,7,8]. Our study replicated the results that the majority of dogs relinquished were unaltered, but we found no difference in gender, income or education levels between respondents surrendering neutered and non-neutered dogs. We did find a significant difference between communities: more dogs in DC came in unaltered compared to NYC (82% DC; 66% NYC). One likely explanation for this could be that there are more accessible and low-cost spaying/neutering options in NYC, and these resources have been available for many years. 

The demographics of the relinquishers in this study are diverse and do not fit the stereotype of a “typical” relinquisher profile that some animal welfare professionals would assume, with lower levels of education and income. The sample in this study included a wide range of income and education. Forty-nine percent of people in NYC and 51% in DC reported incomes of less than $35,000, and 21% of respondents in NYC and 32% in DC reported incomes of over $45,000. The majority of people reported having completed a bachelor’s degree or at least some college (66% NYC, 61% DC), and 3% in NYC and 10% in DC had a graduate or professional degree. It is interesting to note that these samples are representative of the U.S. census data for their communities. 

While most people in NYC lived in an apartment (which is not surprising, as the majority of domiciles are apartments in NYC), 30% of people in DC lived in a single family home. This may suggest that there is a regional component to the housing difficulties experienced by relinquishers. It is also possible that the finding that relinquishers tend to live in rental housing is related to the common type of housing in a particular location rather than any unique characteristic of the people who rent. We found that the majority of households included at least one child aged 12 years or younger (67% NYC, 77% DC). These results, overall, show that a wide variety of people relinquish dogs.

The majority of people said that something had recently changed in their household that contributed to the decision to relinquish their dog, and when asked what had changed, the majority of responses had to do with people or housing issues. Very few were dog-related issues, such as behavior or the cost of caring for the dog. These results suggest that for most people who relinquished their dog in this study, the decision to relinquish was made (at least partially) based on factors unrelated to the dog. By asking what had recently changed, it is also possible that bothersome behavior issues existed, but that those issues, coupled with recent events, pushed people to make a decision to relinquish. Previous studies have highlighted the importance of behavior issues in contributing to relinquishment decisions [8,21]. Our results are consistent with previous research by DiGiacomo *et al*. [14], showing that most people think about the decision for some time, with 51% in NYC and 48% in DC thinking about it for a month or more.

Most people reported exploring other options before going to the shelter. The vast majority of people, 85% in NYC and 69% in DC, reported that they tried something to avoid relinquishing the dog. It is clear, however, that people have a limited awareness of their options. The option most often tried was asking friends or family to take the dog. Interestingly, “friends/family” was also the most commonly reported source of where the person originally obtained the dog, indicating that perhaps dogs are being passed around within families or friends. Of note is the number of people in NYC who tried a helpline, other shelters or rescues. It might be that more people are aware of other shelters and rescues in the NYC area, at least partly because the availability of a helpline is prominently displayed on the “Surrender an Animal” page of the NY ACC website. There is no helpline or relinquishment information posted on the DC website. These results suggest that if options are available and known, people will try them. Providing more programs and services to support people who are struggling with the decision to relinquish their dog has the potential to significantly reduce intake into animal shelters.

Further evidence that providing programs and services to help people keep their dogs could be effective was shown by the fact that the majority of people said something could have helped them keep their dog. Many people wanted to keep their dogs and had clear ideas of what could have helped. Many ideas provided by the respondents, such as temporary pet-friendly housing or boarding, or financial help for dog costs, such as veterinary care or training, could be addressed with targeted programs or services. Related to this is the fact that of the people in NYC who reported having reservations before obtaining their dog, 32% of them had concerns regarding housing, including knowing that their housing did not allow dogs. Sharing information with potential dog owners around possible housing issues, as well as increasing the availability of pet-friendly housing, in general, could help prevent many dogs from entering the shelter system. Simply put, it is often less expensive to cover the cost of a pet deposit than it is to care for the dog in a shelter setting.

When thinking of creating targeted programs and services designed to reduce intake, our results indicate that there is no “one size fits all” solution. While our study focused on just two communities, these communities were both large, urban communities in the North East, and it would not be unreasonable to assume that the reasons for relinquishment would be the same. Of the communities studied here, while similar in many characteristics, they were very different in those that could be key to designing effective interventions. For example, many more people in DC reported that temporary pet-friendly housing or boarding would have helped, indicating the transient nature of their need. It may be that people in DC would benefit from a bridge between moves to enable them to keep their dog. People in both NYC and DC reported the need for more pet-friendly housing, in general, but 26% of people in NYC said they needed more space. While it is difficult to know what respondents really meant by “needing more space”, it may be that pet walking services or day care services could help. Space might have also meant the need to expend energy; certainly, this is a word that could use more probing in future surveys. Other significant differences between the communities include ethnic identity; in DC, the majority of people reported that they were African-American, while in NYC, the majority was Latino/Hispanic. As mentioned, the type of housing was different, with nearly everyone in NYC reporting that they lived in an apartment or condo, while 50% in DC lived in apartments/condos and 30% lived in single family homes. It is likely based on these data that each community may need to create different types of interventions to be effective in that community to reduce the relinquishment of large dogs.

Finally, our results refute a common myth that all people who relinquish their dogs do so without thought or care for the dog. Most people in our study took a long time to think before they relinquished the dog. The majority of people tried something to avoid relinquishing the dog, and they thought it was possible that something could have helped them keep their dog. Poignantly, when asked what they liked best about their dog, most responses were caring and demonstrated attachment. Responses included “dog is like a child to them” or “just loved him” or “he was always excited to see me”. In addition, when asked what they would tell a new adopter, responses included comments like “she is affectionate and lies in bed with you” or “he is a good, loving dog” or “he will make you love him and pet him”. Responses to this question often included specific likes or dislikes of the dog, such as “loves to have his belly rubbed” or “likes to watch TV”. These results strongly suggest that many of those relinquishing still love their dogs, do not want to relinquish them and indicate that if options are available that will allow them to keep their dog, they would consider taking them. 

It is apparent that there are not enough options available and/or people’s knowledge of available services is limited. The development of programs and services, created with careful consideration of each individual community’s needs, as well as a concerted effort to market those services, has the capability to decrease the intake of large dogs into community animal shelters and to save more lives.

## 5. Potential Limitations

Our study has several potential limitations, but is still one of the most in-depth and recent projects regarding pet relinquishment. While the interviewers in both locations were trained in similar ways, their approaches may have differed in subtle ways. For a sensitive topic like this, the interviewer’s approach and tone of voice is critical to obtaining accurate and complete information. Similarly, the lobbies in which the interviews were conducted were not identical and may have contributed to differences in responses. It is also possible that some respondents provided socially acceptable answers rather than accurate responses, despite our attempts to mitigate this issue by interviewing respondents after they had surrendered their pet in an area removed from the surrender counter. We also assured respondents that their responses would not be shared with shelter staff. The interviewers were only present during some times and days at the shelters and were not always able to interview all of the relinquishers on those days, which could influence the types of relinquishers included in this study. The responses of people who were missed or who refused to be interviewed may have differed from those of people that participated in ways that are difficult to know. Another possible limitation was misclassification bias due to our interpretation of the open-ended questions and our categorization of them. All of the authors agreed upon what was included in which category. However, some responses, such a “friendly”, could possibly have been considered by others to be a characteristic of the dog’s personality, rather than a type of interaction with people.

Lastly, this study was conducted in only two shelters, and the relinquishers, their dogs and their issues differed from one another. Therefore, before designing supportive programs for your shelter, conducting a similar survey with a sample of people surrendering large dogs to your shelter could help target support services for your community. 

## Appendix

### Large Dog Relinquishment Survey

Approach all coming in to the intake area that:

‐ Have a dog that appears to be at least 4 months of age
‐ Have a dog that appears to be at least 40 lbs
‐ Have a mother with a litter of puppies (you will interview the owner regarding the mother)

Who to not approach:

‐ Those coming with cats
‐ Those coming with a litter of puppies without the mother

### For people who are visibly upset:

Hi there. I know that this is a very difficult time and I am so sorry that you have to do this. I am doing some research for the ASPCA to determine if there is anything that we might be able to do to help people before they are at this point of needing to bring their pet to a shelter. Would you be able to answer a few questions to help us with our research? We are hopeful that we might be able to prevent other people and animals from being in this situation in the future, and your participation is very important; you will be helping lots of animals who would otherwise come to the shelter and keeping their owners from having to go through this sad experience. This survey is voluntary – it is not a part of the shelter’s intake process and is not required for you to be able to leave your dog here today.

*After survey is complete:* *Thank you for answering my questions. I know that today has been very sad, so I appreciate your taking the time to do this. You are helping a lot of dogs by participating in this research.*

### For people who just seem to be in a hurry/are indifferent:

Hi there. I know that you are in a hurry and that bringing your dog here today must be difficult. I am doing some research for the ASPCA to help us develop programs to keep animals out of shelters. It would be very helpful if you could answer a few questions for us. This will only take about 10 minutes. Although this is voluntary, and is not a part of the shelter’s intake process, your answers are very important and I would really appreciate your participation.

*After survey is complete:* *Thank you for answering my questions. It has been very helpful, and I appreciate your time.*

What is your dog’s name?

1. Does (dog’s name) belong to you or a family member?
  If no, please say: “Thank you for your time. We are conducting a survey in which we need to speak with either the owner of the pet or a family member.”
  If yes, ask: “Will you be able to answer questions about this dog’s history in the home?”
2. Have you owned (dog’s name) for less than 2 weeks?
  If they have had the dog for less than 2 weeks, please say: “Thank you for your time. We are conducting a survey in which we need for the dog to have been in the home for at least 2 weeks. We don’t have any more questions for you at this time.”
3. How many other dogs are in the household from which (dog’s name) came? __________________
4. Is (dog’s name)
  - a male
  - a female
5. How old is (dog’s name)?
  - < 3 months
  - 3 to < 6 mo
  - 6 to < 9 mo
  - 9 to < 12 mo
  - 1 to < 2 yrs
  - 2 to < 4 yrs
  - 4 to < 5 yrs
  - 5 to < 10 yrs
  - 10 to < 15 yrs
  - 15 years plus
  - Don’t know
6. Is (dog’s name) spayed/neutered?
  - YES, approximately how old was s/he? ________________________________
  - NO
  - DON’T KNOW
7. Is (dog’s name) a purebred?
  - If yes, what breed? ________________________________________________
    *Interviewer: Please note if the dog appears to be a bully breed or bully breed mix to you* Y / N
  - No
  - Don’t know
8. Where did you obtain (dog’s name)?
  - Breeder
  - Friend
  - Shelter
  - Neighbor
  - Born in home
  - Found as stray
  - Pet store
  - Other ____________________________________________________________
9. About how much did it cost to get (dog’s name)?
  - $0
  - $0.01 to < $100
  - $100 to < $200
  - $200 to < $300
  - $300 to < $400
  - $400 plus
  - Don’t know
10. Think back. When did you get (dog’s name)? (probe for year and month, if possible)
  __________________________________________________________________________
11. Why did you get/keep a dog at that time? Circle all that apply.
  - Companionship for children
  - Companionship for adult(s)
  - Companionship for a dog already in the household
  - Teach the children responsibility
  - To show him/her off to friends
  - For protection of my residence, me or family members
  - To help a friend/acquaintance who couldn’t keep the dog
  - To save his/her life
  - S/he was cute and available or other spur of the moment reason
  - S/he was a gift
  - Other, please describe the reasons/circumstances _________________________________________________
12. What characteristics did (dog’s name) have that led you to select or agree to keep him/her for your dog (as opposed to another dog)?
  _______________________________________________________________________
13. Did you have any reservations or worries about getting him/her back then? Please describe.
  ________________________________________________________________________
14. Thinking back to those first few months after you first got (dog’s name), can you tell me what you liked best about him/her?
  ________________________________________________________________________
15. Has (dog’s name) been to the vet in the last year (or since you have owned him/her if you have owned for less than a year)? If yes, how many times and for what?
  ___________________________________________________________________
16. Has anything changed recently in your household (e.g., family moved, illness, or death in family) that contributed to the decision to bring (dog’s name) to the shelter now?
  - YES, please describe
    _______________________________________________________________________
  - NO
    Follow-up to the specific reason: *i.e.*, if it is a housing problem, did the landlord suddenly change his mind about allowing the dog, if you are moving for a place that doesn’t take the dog, could you not find anywhere that would allow a dog or was it cost prohibitive to bring the dog, *etc*.
17. Tell me about why you are bringing (dog’s name) here today (Other than what we discussed above)? Collect up to 5 reasons.________________________________________________
18. Were there other options that you or the family explored that led to dead ends before making the decision to come here?
  - YES, please describe _____________________________________________________________________
  - NO
19. Who was involved in making the decision to bring (dog’s name) to the shelter?
  - Only I was
  - My spouse and I
  - The entire family (including children)
  - My parents
  - My parents in consultation with me
  - Other, please describe ______________________________________________________________________
20. How long have you (or the family) been thinking about bringing (dog’s name) to the shelter? _______________________________________________________________________
21. We are trying to provide temporary housing, make available resources, help with behavior issues or other programs that would help people keep their dogs (if possible).
  Thinking back, do you think that there is anything (e.g., temporary housing, money to get more dog training or medical care) that could have helped you (your family) keep (dog’s name)?
    - YES
    - NO
      If YES, what do you think might have helped?
      ________________________________________________________________________
22. If I was to be this dog’s new adopter, what would you tell me about him/her?
  __________________________________________________________________________
23. In which range is the YEARLY INCOME of your household?
  - Less than $12, 500
  - $12,500-$19,999
  - $20,000-$27,499
  - $27,500-$34,999
  - $35,000-$44,999
  - $45,000-$74,999
  - $75,000-$124,999
  - More than $124,999
24. We may want to follow up with a subset of folks we interviewed today to get more detailed information about your experience, would you be willing to share your contact information with us?
  If yes, info.: _________________________________________________________
25. What is your cultural or ethnic identity?
  - Hispanic/Latino
  - White/Caucasian
  - African-American/Black
  - Asian
  - Native American/American Indian
  - Other (please specify)_______________
26. Thinking of the person in your household who has completed the most school, what is the highest grade level achieved by that person?
  - Grade 8 or less
  - Some high school
  - Completed high school or GED
  - Some college
  - Bachelor’s Degree
  - College work beyond a Bachelor’s Degree (Graduate or professional degree)
  - Other, please describe ________________________________
27. How many people of the following ages currently live in your household?
  ______ number of children less than 5 years old
  ______number of children 5 to 12 years old
  ______ number of children 13 to 19 years old
  ______ number of adults 20 to 64 years old
  ______ number of adults 65 years old or older
28. What type of dwelling do you currently live in?
  - Apartment on condominium
  - Mobile home or trailer
  - Single family house
  - Duplex/attached house
  - Other, please describe __________________________

*Thank you for your cooperation and help!*
